# An experience of subglottic airway foreign body removal in a patient under tracheal intubation: a case report

**DOI:** 10.1186/s40981-020-00382-z

**Published:** 2020-10-04

**Authors:** Shiori Tanaka, Keisuke Yoshida, Kenichi Muramatsu, Shigeki Yamagishi, Shinju Obara, Kazuhiro Watanabe

**Affiliations:** 1 Department of Anesthesiology, Aidu Chuo Hospital, 1-1, Tsuruga-machi, Aizuwakamatsu, Fukushima, 965-8611 Japan; 2 Department of Anesthesiology, Fukushima Medical University, 1, Hikariga-oka, Fukushima, Fukushima 960-1297 Japan; 3 Department of Cardiovascular Surgery, Aidu Chuo Hospital, 1-1, Tsuruga-machi, Aizuwakamatsu, Fukushima, 965-8611 Japan; 4 Department of Pulmonology, Aidu Chuo Hospital, 1-1, Tsuruga-machi, Aizuwakamatsu, Fukushima, 965-8611 Japan

**Keywords:** Airway foreign body, Under intubation, Subglottic airway foreign body

## Abstract

**Background:**

Removal of an airway foreign body is challenging to anesthesiologists. We report successful removal of an extremely rare foreign body between a tracheal tube and the trachea in patients under tracheal intubation.

**Case presentation:**

A 57-year-old male received total aortic arch replacement and postoperative mechanical ventilation. An airway foreign body was detected just below the glottis, outside the tracheal tube during mechanical ventilation after surgery in the intensive care unit. Before the removal procedure, we planned multiple strategies to cope with unexpected airway and breathing troubles. As a result, the foreign body was successfully removed orally by using a bronchial fiber, without extubation of the tracheal tube, under general anesthesia with dexmedetomidine and ketamine.

**Conclusions:**

We reported the successful removal of a foreign body in the subglottic airway of a patient under tracheal intubation.

## Background

Despite several reports regarding airway foreign bodies in the subglottic region [1–3], there have been no reports of those located between a tracheal tube and the trachea in patients under mechanical ventilation. We here report successful removal of a subglottic airway foreign body in a patient under tracheal intubation.

## Case presentation

A 57-year-old male (height 166 cm, weight 92 kg, body mass index 33.4 kg/m^2^) received total aortic arch replacement for acute type A aortic dissection. His past medical histories included right nephrectomy for right kidney cancer and hypertension. The aortic replacement was performed under general anesthesia, which was induced with propofol (target-controlled infusion [TCI] 6.0 μg/mL), fentanyl 0.5 mg, and rocuronium 100 mg. Thereafter, a cuffed tracheal tube with an inner diameter of 8.0 mm was orally intubated and fixed at 22 cm at the corner of the mouth. There were no particular problems, including tooth damage during tracheal intubation. Anesthesia was maintained with propofol (TCI 2.0–2.2 μg/mL, total 4860 mg), remifentanil, fentanyl (total 2.5 mg), and rocuronium. The operative time was 532 min, and the anesthesia time was 638 min.

After the surgery, the patient was transferred to the intensive care unit (ICU) with artificial respiration, and dexmedetomidine 0.43 μg/kg/h and fentanyl 30 μg/h were administered for sedation and analgesia. On the first postoperative day (POD 1), an extreme elevation of creatine kinase (CK, 3,730 U/L) and a 39 °C fever of uncertain etiology were observed. We suspected propofol infusion syndrome (PRIS), malignant hyperthermia (MH), neuroleptic malignant syndrome, drug-induced rhabdomyolysis, and/or serotonin syndrome; therefore, we ceased administration of any potentially related drugs (propofol, piperacillin/tazobactam, fentanyl) and started administration of midazolam and dexmedetomidine instead. Although the CK value peaked at 45,288 U/L on POD 7 after gradual increase from POD 1, it was decreased after POD 8 due to the dantrolene 40 mg administrated on POD 7. In addition, a decrease in renal function (estimated glomerular filtration rate: 17.6 mL/min/1.73 m^2^) was observed postoperatively; thus, continuous hemodiafiltration (CHDF) was performed from POD 2. Moreover, mechanical ventilation had been continued because of poor oxygenation due to pneumonia since surgery. We did not perform tracheostomy because of the high possibility of mediastinitis due to the protruding sternal wires and possible difficulty of hemostasis by anticoagulants for CHDF, in spite of long-term mechanical ventilation.

**Fig. 1:**
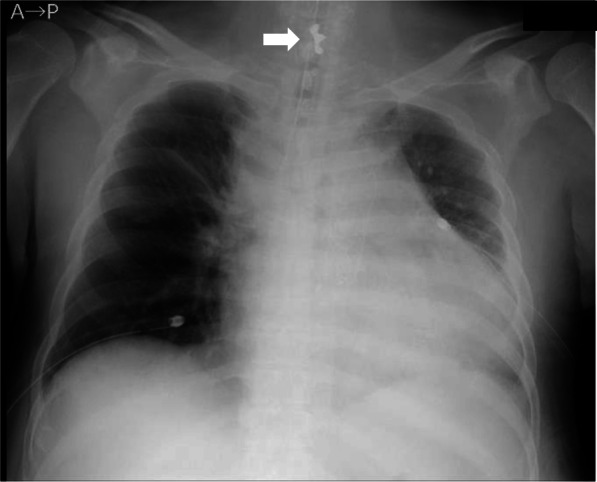
A chest radiograph taken on POD 34. A foreign body was confirmed at the midline of the neck (arrow). POD, postoperative day

**Fig. 2:**
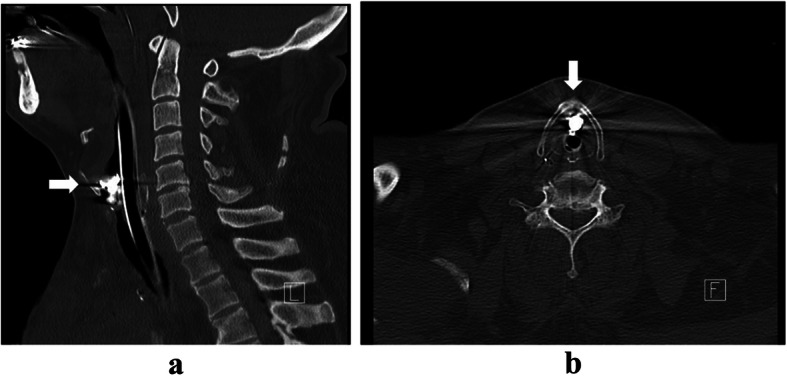
Simple CT scans on POD 34. **a** Sagittal section. An airway foreign body (arrow) was found on the ventral side of the tracheal tube at the level of the 5th and 6th cervical vertebrae. **b** Horizontal section. An airway foreign body (arrow) with metal artifacts was observed. CT, computed tomography; POD, postoperative day

A chest X-ray on POD 34 revealed an abnormal shadow near the larynx (Fig. 1). Review of previous X-ray showed the same shadow in the hypopharynx on POD 19, although it was not identified immediately after arrival in the ICU. A loss of a front tooth was found on POD 22; however, a foreign substance was not confirmed in the oral cavity or on the abdominal X-ray, and the patient was placed under observation. A simple computed tomography (CT) scan on POD 34 showed a shadow with metal artifacts, indicating a crown bridge, outside of the tracheal tube, inside the trachea, and just under the glottis (Fig. 2). Upper esophageal gastrointestinal endoscopy performed on POD 34 also revealed tooth fragments in the stomach. The pharynx and larynx were examined at bedside using McGRATH^TM^MAC video laryngoscope (Medtronic, MN, USA); however, no foreign matter was found. Observation from the inside of the tracheal tube using the bronchial fiber revealed a foreign body in the trachea just below the glottis, outside (ventral side) the tracheal tube. We therefore considered that the removal of the foreign body in a well-equipped environment would be more suitable than in the ICU and that the foreign body would not cause airway obstruction immediately.

Removal of the airway foreign body was performed in the operating room on POD 35. Preoperative ventilator settings were pressure support (PS) mode, an inspiratory oxygen concentration of 60%, a PS of 8 cmH_2_O, a positive end expiratory pressure of 5 cmH_2_O, and an arterial oxygen partial pressure of 274 mmHg. His consciousness level was E4VtM5 on the Glasgow Coma Scale. The CHDF and administration of heparin were interrupted 90 min before entering the operating room. For the procedure, three anesthesiologists, one thoracic surgeon (bronchoscopy operator), one cardiac surgeon (attending physician), and three nurses were convened, and a difficult airway management (DAM) cart was prepared in the operating room.

**Fig. 3:**
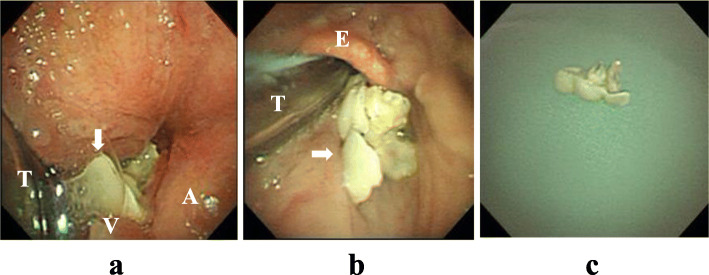
Photographs taken with a bronchial fiber. **a** A foreign body (arrow) was located between the vocal cord (V) and the arytenoid cartilage (A) outside of the tracheal tube (T). **b** The foreign body during the removal procedure. The foreign body (arrow) was pulled up between the tracheal tube (T) and epiglottis (E). **c** A removed crown bridge, whose backing metal was covered with resin. It consisted of three connected teeth with a pointed edge

The patient entered the operating room while maintaining spontaneous breathing. After administration of atropine 0.5 mg, continuous administration of dexmedetomidine 0.22–0.89 μg/kg/h was started under standard monitoring as well as neuromuscular monitoring. In addition, ketamine 10 mg was administered seven times in total while observing the capnogram and the patient’s condition. Then, his respiratory rate became 12 breath/min, and he was sedated (Richmond Agitation-Sedation Scale score of − 2 to − 3). A total of 5 mL of 4% lidocaine was sprayed into the pharynx and larynx for local anesthesia via a Jackson’s spray. With the trachea intubated, the larynx was visualized using a blade size 4 of McGRATH^TM^MAC, and a bronchial fiber (OLYMPUS BF TYPE 1T260, tip outer diameter 5.9 mm, Olympus Corporation, Tokyo, Japan) was inserted into the oral cavity to observe the larynx. No foreign body was confirmed in the pharynx and larynx; however, when the bronchial fiber was inserted into the glottis from the gap between the glottis and the cuff of the tracheal tube, a part of the foreign body appeared and disappeared between the arytenoid cartilage and the vocal cord according to the patient’s breathing (Fig. 3a). Although we could not confirm the whole image of the foreign body, we were able to confirm that it was mobile. At this point, hiccups, cough, and/or gag reflex occurred and it became difficult to secure a field of view with the fiber. We judged that the foreign body could be removed via an oral approach using the bronchial fiber while maintaining intubation. Thus, rocuronium was administered to eliminate spontaneous breathing and body movement, and mechanical ventilation was started. After that, grasping forceps were inserted via the bronchial fiber, and the foreign body was pulled up to the pharynx. Then, it was removed using Magill forceps (Fig. 3b, c). Following the administration of sugammadex 200 mg, the patient’s respiratory condition returned to the preoperative level, and he was returned to the ICU. The surgery time was 35 min, the anesthesia time was 51 min, the lowest percutaneous oxygen saturation during the procedure was 93%, and there was no bleeding associated with the removal procedure.

## Discussion

In patients under tracheal intubation, it is extremely rare for an airway foreign body to be detected between the tracheal tube and the trachea. To the best of our knowledge, the current case report is the first.

A foreign body in the trachea becomes a source of infection if it is left for a long period, and if it falls into the bronchi, it leads to deterioration of the patient’s respiratory status. Furthermore, a foreign body may perforate the trachea, leading to mediastinal emphysema [4]; therefore, it should be removed immediately after detection. The gold standard for removal of adult airway foreign bodies is a procedure using a bronchial fiber [5]. However, in the present case, we decided that it was necessary to make a detailed plan in advance, because of the following problems: (1) the patient was under tracheal intubation; (2) the foreign body was located just below the glottis, which might require moving the foreign body to the bronchus first as difficulties in the control of the bronchial fiber were anticipated; (3) insufficient oxygenation due to pneumonia; (4) difficulties in choosing tracheostomy; (5) risk of airway edema due to long-term tracheal intubation; (6) bleeding risk due to the use of anticoagulants; and (7) necessity to avoid the use of propofol and MH-associated drugs for the procedure.

Prior to the removal procedure, we planned the following procedures: (1) prepare a DAM cart and a bronchial fiber, considering the possibility of re-intubation and deterioration of respiratory condition after extubation because of having more than one month of intubation and possible risk of difficult airway; (2) mark the position of the cricothyroid ligament for emergency surgical airway preparation; (3) insert the bronchial fiber orally to confirm the position and mobility of the foreign body; (4) if it is difficult to confirm the above and remove the foreign body via oral approach, extubate with a tube exchanger left in place for re-intubation; (5) after extubation, perform the foreign body removal under spontaneous breathing while maintaining oxygenation with high-flow nasal cannula [6]; (6) if upper airway obstruction occurs, insert a supraglottic airway device and insert the bronchial fiber into the device; (7) if oxygenation cannot be maintained during the removal procedure or airway obstruction is caused by the foreign body, drop the foreign body to the bronchi and re-intubate, then retry the removal using the bronchial fiber inserted into the tracheal tube; and (8) if the retry fails, terminate the procedure, and reconsider the removal plan. Although the use of muscle relaxants was not included in the original plan, rocuronium was prepared for possible use. As a result, in the present case, the airway foreign body was successfully removed using a bronchial fiber by intraoral approach under general anesthesia with dexmedetomidine and ketamine, without extubation of the tracheal tube.

Several strategies other than the aforementioned plan were also considered. Extracorporeal membrane oxygenation (ECMO) is one of the choices as reported previously [7]. However, blood access was limited because of CHDF in the present case. In addition, his respiratory condition had improved to such a level that a spontaneous breathing trial could be performed. Thus, we determined that foreign body removal under spontaneous breathing after extubation was possible for a short time and did not use ECMO.

Regarding anesthesia, there is no fixed rule for the removal of foreign bodies from the respiratory tract, and to the best of our knowledge, it is not related to adverse events under either spontaneous or controlled breathing [8]. In the present case, since the patient was suspected to have PRIS and/or MH (although a definitive diagnosis had not been made), we could not use propofol or MH-associated drugs such as sevoflurane [9, 10] and used a combination of dexmedetomidine and ketamine. Because this combination is more likely to preserve spontaneous breathing and has a higher analgesic effect [11], the removal procedure could be performed under spontaneous breathing even if extubation was performed. Although there are many other choices such as remifentanil and midazolam, it would be safe to select anesthetics with which anesthesiologists are familiar.

In addition, the use of muscle relaxants was not included in our original plan because of the possibility to perform the removal under spontaneous breathing after extubation. This is because the patient had several risk factors of difficult airway [12] such as obesity, thick neck, limited thyromental distance, and more than one month of intubation. However, cough and gag reflex may have moved the foreign body into the distal/proximal side during spontaneous breathing; therefore, whether to use muscle relaxants should be decided depending on the situation.

Meanwhile, there are some points to note. In the present case, there was a time lag between the patient reporting losing his tooth and its eventual detection in the glottal region. The delay of the detection might be associated with the following: the assumption that an airway foreign body outside the tracheal tube would not occur during tracheal intubation, and the pharynx was not included in all chest X-ray images. Fortunately, this time lag allowed his respiratory condition to improve to a level where a short-term procedure under spontaneous breathing was selectable. Additionally, when interpreting a chest X-ray image of an intubated patient, we tend to pay most attention to the lung field. However, we now know it is important to interpret not only the lung field but also the entire image to prevent overlooking. Moreover, although the mechanism of the foreign body entering the trachea during intubation was unknown in the present case, a thorough search is necessary to determine the location when tooth loss is reported in patients.

## Conclusions

We here report the successful removal of a foreign body in the subglottic airway of a patient under tracheal intubation. Our case highlights the importance of planning multiple strategies in order to cope with unexpected airway and breathing troubles.

## Data Availability

Not applicable.

## Abbreviations

TCI: Target-controlled infusion
ICU: Intensive care unit
POD: Postoperative day
CK: Creatine kinase
PRIS: Propofol infusion syndrome
MH: Malignant hyperthermia
CHDF: Continuous hemodiafiltration
CT: Computed tomography
PS: Pressure support
DAM: Difficult airway management
ECMO: Extracorporeal membrane oxygenation
